# Efficiency and Improved Profitability of Insect-Based Aquafeeds for Farming Nile Tilapia Fish (*Oreochromis niloticus* L.)

**DOI:** 10.3390/ani11092599

**Published:** 2021-09-04

**Authors:** Moses N. Wachira, Isaac M. Osuga, Jonathan M. Munguti, Mary K. Ambula, Sevgan Subramanian, Chrysantus M. Tanga

**Affiliations:** 1 International Centre of Insect Physiology and Ecology, P.O. Box 30772-00100 Nairobi, Kenya; isaac.osuga@jkuat.ac.ke (I.M.O.); ssubramania@icipe.org (S.S.); ctanga@icipe.org (C.M.T.); 2 Department of Animal Sciences, Egerton University, P.O. Box 536-20115 Njoro, Kenya; m.ambula@egerton.ac.ke; 3 Department of Animal Science, Jomo Kenyatta University of Agriculture and Technology, P.O. Box 6200-00200 Nairobi, Kenya; 4 Kenya Marine and Fisheries Research Institute, P.O. Box 451-10230 Sagana, Kenya; jmunguti2000@gmail.com

**Keywords:** aquaculture, insect meal, fishmeal, fortified aquafeed, earthen pond, Kenya

## Abstract

**Simple Summary:**

Fish farming in sub-Saharan Africa remains a source of livelihood for many househlods, but increased productivity is severely constrained by the high cost of fish feeds through the use fishmeal (FM) which is usually not easily available and when available is expensive. Therefore, this study evaluated the suitability of black soldier fly larvae meal (BM) as an alternative protein to FM. Four diet types were tested: control (100% FM; 0% BM), BM33 (67% FM; 33% BM), BM67 (33% FM; 67% BM) and BM100 (0% FM; 100% BM). The experiment was conducted for 20 weeks. The average daily feed intake and body weight gain of the fish were affected by the treatment diet. However, the survival rate and feed conversion ratio were not affected by the diet. The fish fed on diet BM33 had a 14.4% increase in weight gain compared to that of the control diet. Return on investment and the cost–benefit ratio were similar for various diets, suggesting that BM can be a suitable and cost-equivalent dietary protein substitute of FM in aquafeed for growing tilapia fish in earthen ponds for the market.

**Abstract:**

In Sub-Saharan Africa, Nile tilapia (*Oreochromis niloticus* L.) make up over 80% of aquaculture production. However, the local aquaculture farmers are restricted by the unavailability and expensive cost of formulated rations. To reduce reliance on the scarce and expensive fishmeal used in fish feeds, alternative insect protein has been successfully utilized in many aquafeeds. However, data on the influence of insect-based feed on the growth and economic benefit of feeding tilapia with the emerging insect-based diet are scanty. This study investigated the effect of partially and completely substituting fishmeal with black soldier fly larval meal (BM) on growth and economic parameters of tilapia. The *O. niloticus* was fed a standard commercial diet as a control (100% FM; 0% BM), BM33 (67% FM; 33% BM), BM67 (33% FM; 67% BM) and BM100 (0% FM; 100% BM) for 20 weeks in randomly assigned cages mounted in an 800 m^2^ earthen pond. Results from this study showed that diet type significantly (*p* < 0.05) affected the feed intake of the fish as well as weight gain. The feed conversion ratio and survival rate of *O. niloticus* did not vary across the different diets. Fish fed Diet_1_ had a 15% increase in weight when compared to fish fed the control diet. Return on investment and the cost–benefit ratio was similar across the diets, suggesting that BM is a suitable and cost-equivalent dietary supplement of FM up to 100% in aquafeed for growing tilapia fish in earthen ponds for the market.

## 1. Introduction

The global human population is rising at a rate of 75 million people annually, and by the year 2050, it is estimated to be 9.7 billion people from the current 7.7 billion people especially in the low-income countries and more so in sub-Saharan Africa (SSA) [1]. This population growth coupled with an increase in incomes, shifts in dietary patterns and urbanization is expected to double the demand for animal protein by 60% [2]. Aquaculture production has a huge potential for expansion for increased fish supply, which is an important high-quality source of animal protein for human food [3]. Fish production contributes significantly to the supply of animal protein for human consumption. For instance, fish consumption per capita was shown to increase to 20.5 kg in 2017 from 9.0 kg in 1961, with a 15% per year average growth rate [2]. In terms of aquaculture production in SSA, there is a projected increase of 0.231 million tonnes to 0.464 million tonnes from 2007 to 2030 [3], with tilapia (*Oreochromis niloticus* L.), carp (*Cyprinus carpio* L.) and catfish (*Clarias gariepinus* Burchell) being the main cultured fish species [4,5,6]. To achieve this production increase, it is important to enhance the efficiency of production while maintaining environmental sustainability [7]. The choice of ingredients and diet formulation for fish greatly determines the impact of the aquaculture on the environment. Traditionally, fishmeal (FM) and fish oil as well as plant-based feeds such as soybean have been used to formulate fish diets [8]. However, marine stocks harvested from the wild for fishmeal production are decreasing and from ecologic and economic points of view, the plant-based protein sources are no longer sustainable [9]. Given the aforementioned, there is need for research to come up with feed ration formulations for fish with alternative protein sources [10].

Insect meals have been proposed as promising and sustainable protein sources to fishmeal in livestock diets [8,11,12]. Insect production requires six times less feed than conventional livestock to produce the same amount of proteins [13]. In addition, the greenhouse gas emission from the insects is much less compared to the conventional livestock in the production of food/feed. Further, the insects can be mass produced using organic waste streams [11], which cannot be included directly in livestock and fish feeds [14]. In the wild, fish consume insects. At the bottom of water bodies, omnivorous fish species prey on insects while carnivorous fish species consume juvenile life stages of insects before switching to adult insect-based diets [15]. The sustainable utilization of insects as feed has been attributed to their relatively high amounts of protein, fatty acids, energy, well-balanced amino acids and minerals (sodium, iron, potassium and zinc) [16]. 

Insects such as the housefly, mealworm, grasshopper, black soldier fly and cricket have been identified as good alternatives to fishmeal. Black soldier fly larvae (BSFL) have the highest potential being the most effective converter of organic waste into valuable biomass of high protein value [14]. The BSFL usually grow on pig, cattle manure, poultry manure, fish offal, vegetables, coffee bean pulp and others [17]. The adult black soldier flies do not eat [17]; thus, the larvae must accumulate a large fat body (energy) and protein necessary to go through the larval stage and survive during the adult stage, mate and lay eggs [18]. 

Several research studies with fish fed diets with varying inclusion levels of black soldier fly larvae meal (BM) have reported good growth performances similar to those from fish fed on common protein sources such as FM and soybean. Fingerlings of Channel catfish (*Ictalurus punctatus* Rafinesque) had a similar weight gain when subjected to diets supplemented with up to 30% of full-fat black soldier fly larvae meal (BM) [19]. Fishmeal substitution with full-fat BM with varying levels of up to 25% in diets for the rainbow trout, *Oncorhynchus mykiss* Walbaum, has been shown not to have any negative effect on weight gain [20]. A growth experiment with treatment diets containing BM at the inclusion rate of 18% and 36% prepupae showed a similar performance to rainbow trout fed a control diet containing anchovy meal [21]. Therefore, this study aimed to evaluate the effects of partial or complete substitution of FM with BM on the growth performance of *O. niloticus* to market size in earthen ponds. The economics of the formulated rations were also established.

## 2. Materials and Methods

**Research facilities:** Research activities were carried out in the Sagana station of the Kenya Marine and Fisheries Research Institute (KMFRI) in Central Kenya. The site is situated at an altitude of 1230 m above sea level, within the latitude of 0′39 S and longitude of 37′12 E. The ambient temperatures range between 27 °C and 32 °C and an annual rainfall of 1138 mm.

**Experimental diets:** The ingredients required to formulate the various experimental diets for the feeding experiment except BM were purchased from local Agrovet suppliers at Sagana Town, Kenya (Table 1). Black soldier fly larvae were sourced from the production facility located at the International Centre of Insect Physiology and Ecology (*icipe*), Nairobi, Kenya. These insects were reared on a brewer’s spent grains acquired from East African Breweries Limited, Nairobi, Kenya. Optimal rearing of BSF was undertaken at 28 ± 1 °C, relative humidity of 60–70% and photoperiod of 12 h light, 12 h dark. Larvae were harvested at the 5th instar stage, cleaned and sterilized in a water bath at 84 °C for 10 min. Thereafter, the larvae were dried through a commercial oven (CT-C-III Series Hot Air Circulating Drying Oven, Henan Forchen Machinery Co., Ltd., Henan, China) at 120 °C within a time period of 2 h 30 min. The dried insect products were ground and mixed with other ingredients (maize germ, wheat pollard and fishmeal) to form the four nutrient-balanced diets. The experimental diets were constituted of partially and completely substituting FM with BM: control (100% FM; 0% BM), BM33 (67% FM; 33% BM), BM67 (33% FM; 67% BM) and BM100 (0% FM; 100% BM). The diets were formulated to meet the optimum feeding standards for tilapia fish [22]. Because the nutritional content of BM was different from that of FM, the substitution levels of FM with BM were carefully carried out to ensure that inclusion levels of some other dietary ingredient (wheat pollard) were adequate. The proximate composition of the various ingredients was conducted, and the results were used as the basis for the feed formulation. The ingredients were milled and after formulation, were hand mixed into a homogenous blend. The formulations were pelletized by adding warm water to the diets at a rate of 5% inclusion and mixing thoroughly to achieve an appropriate consistency before being pelletized using a 2 mm meat mincing machine. The pellets were properly dried and stored in airtight dark bags for further analyses and experimentation.

**Experimental design:** Twelve (12) cages measuring 1.5 m × 3 m × 1 m, which had been mounted in a large earthen pond measuring 40 m × 20 m, were used in the experiment. A 3 m distance was maintained between the cages within the earthen pond. The earthen pond had been stabilized by liming it with calcium carbonate and left to dry for two weeks before filling it with water up to a depth of 1 m. The water was sourced from a diversion from a nearby river in Sagana. Three hundred and sixty (360) male Nile tilapia fingerlings were selected, and their body weight measured (35 ± 2 g) before commencement of the experiments. At the start of experiment, 30 fingerlings were allocated to each cage using a completely randomized design. The fingerlings were provided four diet types, and each diet was replicated three times. After stocking, the fingerlings were subjected to the control diet for a period of two weeks for acclimatization to the experimental conditions. When the adaptation phase was completed, the starting weight of the fish was taken before the feeding trials were initiated. Tilapia fingerlings were fed twice a day at 09:00 h and 03:00 h at 3% body weight. The feeding experiment was carried out for a period of 20 weeks.

**Growth performance and data calculations:** During the 20-week data collection period, weighing of the fish was conducted every two weeks to monitor the growth trends. The quantity of feed provided to the fish was revised every 2 weeks based on the fish body weights. Mortality was recorded throughout the experimental phase. The growth performance including daily weight gain, survival rate and feed conversion rate were calculated. The feed conversion rate was computed as a function or ratio of the daily feed intake in grams (g) divided by the increase in fish weight (g). The survival rate was estimated based on the total number of fish at the end of the experiment divided by the fish stocking population at the beginning of the experiment multiplied by 100.

**Water parameters and chemical analysis:** Water parameters including the pH, conductivity, temperatures (°C), total dissolved solids, salinity, phosphate (milligram per liter (mg/L)), ammonia (mg/L) and nitrates (mg/L) were monitored on a weekly basis with a multi-parameter water quality meter (model H19828, Hanna Instruments Ltd., Chicago, IL, USA). The proximate composition of the experimental diets and the feed ingredients were performed according to the protocol described by Association of Official Analytical Chemist [23]. Moisture content was evaluated based on weight loss during the drying process at 135 °C for 2 h. The dry matter (DM) was calculated as 100 minus moisture content. The ash composition of the samples was established by incinerating the sample overnight at 550 °C. Fat content was estimated using di-ethyl ether as the solvent in a Velp solvent extractor (SER 148/6) (VELP Scientifica, Usmate Velate, Italy). The Kjeldahl method was used to determine the nitrogen content, which was then multiplied by the factor 6.25 to obtain crude protein (CP) content. The acid detergent fibre (ADF) and neutral detergent fibre (NDF) were analyzed using a Velp fibre analyzer (FIWE 6) (VELP Scientifica, Usmate Velate, Italy) [24]

**Economic analysis:** Economic analysis was conducted by applying two indices: Cost Benefit Analysis (CBA) and Return on Investment (RoI). Production costs included variables such as feeds and labor, but only the cost of feeds was used in the calculations of the economic analysis; the labor was assumed to be the same and constant among all the treatments. The ingredient prices in Kenya at the time when the experiment was conducted were used to calculate the feed costs based on the ingredient proportions included in the treatment diets. The revenue generated from the sale of fish produced was calculated as total revenue generated. This ratio of generated revenue and cost represented the cost benefit ratio (CBR). The CBR value above one (>1) implied that the benefits exceeded the costs and vice versa. Return on Investment (RoI) is an indicator of the gain/loss accrued from the inputs in relation to the amount of money invested. The RoI was calculated as the gross profit margin divided by the costs expressed as a percentage [25].

**Statistical analysis:** Data were analyzed using the Statistical Analysis System (SAS, version 9.1) (SAS Institute Inc., Cary, NC, USA) software. One-way analysis of variance (ANOVA) was applied to determine the effect of different diets on the growth performance parameters such as initial weight, final live weight, daily weight gain and feed intake of Nile tilapia and other calculated indices. Bon–Tukey’s multiple comparison test was used to evaluate the difference between means at *p* < 0.05.

## 3. Results and Discussion

The mean values of parameters of water quality measured in the study are shown in Table 2. The pH in the pond ranged from 8.4 to 9.0 with an average of 8.9. The conductivity range was narrow with a mean of 108. The average water temperature within the pond was 26.8 °C. The mean value of the total dissolved solids was 53.8, while the ammonia level was 0.1 mg/L. Water quality parameters are crucial for both the survival and optimum growth of Nile tilapia. These environmental factors usually govern feed consumption, growth and survival of tilapia fish. It is generally believed that the suitable range of water quality parameters ensures the better management of aquatic organisms as well as the aquatic environment [26]. Profitable Nile tilapia farming requires a regular management of water quality for maintaining a suitable environment and to maximize their production. In the current study, all the parameters of water quality monitored throughout the experimental period was within an acceptable range for optimum performance of Nile tilapia [26,27,28].

The results of proximate analysis of the diets are shown in Table 3. No significant differences (*p* > 0.05) were observed across the various diets, except crude fats measured as ether extract.

The dry matter was similar across the various diets. The CP values of the different experimental rations ranged from 28.2 to 29.4%. The four diets had comparable crude protein levels. The optimum protein requirements for young tilapia fish vary from 32 to 50%, while that for adult fish they are between 25 to 30% [22]. These results demonstrate that BM can replace FM in tilapia fish diets without compromising on the protein levels of the diets required for adequate tilapia fish nutrition. The ether extracts (lipids) in the diets were observed to increase with increasing inclusion of BM in the fish diets. Similar to this study, the observation of increasing lipids contents in the diets with increasing BM inclusion has also been reported [29]. The lipid content of the BM ranges from 27 to 37% of DM [14], with the variation being due to the different substrates used in the larvae production. Diets containing a higher inclusion of the BM recorded relatively higher lipid levels since the larvae have higher lipid contents. The optimal lipid dietary requirement for tilapia fish is 5 to 12% [30]. Diet (BM100 (0% FM; 100% BM) had the highest level of lipids (14.4%). However, lipids are usually a preferable energy source to carbohydrates in fish feeds [31]. The fibre levels as measured by NDF and ADF were generally low for all the diets. These fibre levels were comparably similar to that reported in other studies [32]. In studies with channel catfish, the feed consumption was maximum at a NDF content of 19.1% DM, while the maximum tolerable level for NDF which does not affect performance was found to be 24.9% DM [33]. The fibre levels in the current study are within these ranges. Further, in diets with BM, it is anticipated that the larvae exoskeleton which is well-known to contain chitin might have contributed to the increased fibre content of the diets. Therefore, the fibre (chitin) is likely to offer other beneficial effects to the fish such as boosting the immunity of the fish. Ash content of the diets decreased with increasing BM inclusion in the diets, which is in line with the report by Rana et al. [29], who showed that the ash content of dehydrated BM was 5.08%. 

**Figure 1:**
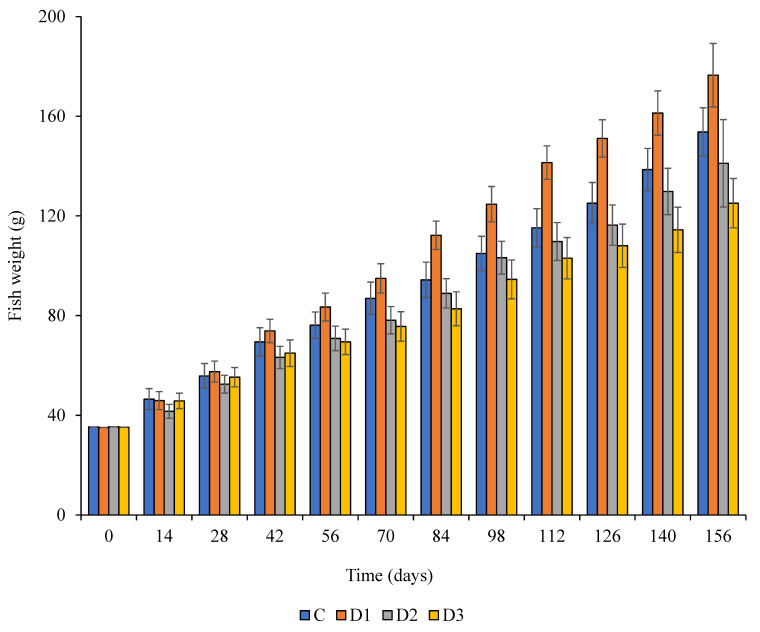
Average weight gain of fish under the four different dietary treatments over a period of 156 days; Control (**C**)—(100% FM; 0% BM); BM33 (**D1**)—(67% FM; 33% BM); BM67 (**D2**)—(33% FM; 67% BM) and BM100 (**D3**)—(0% FM; 100% BM).

The results of partially and complete substitution of FM by BM on the growth of Nile tilapia fish are presented in Table 4. Feed intake, final body weight, daily increase in weight were observed to vary significantly (*p* < 0.05) for the various dietary treatments. The daily weight gain for fish consuming BM33 was statistically different (*p* = 0.01) to that of other diets, while the daily increase in weight was statistically similar (*p* ≥ 0.05) for fish consuming the control diet and BM67 diet. Feed intake was significantly higher (*p* < 0.05) for the fish consuming BM33 followed by the control diet. No significant differences (*p* > 0.05) in feed intake were observed for the fish consuming diets BM67 and BM100. The feed conversion ratio was best in fish fed diet BM33 and high for those fed diet BM100. Survival rates of the fish were not statistically different (*p* = 0.88) for fish fed on all the dietary treatments. The trends in daily weight gain throughout the experiment are illustrated in Figure 1. The weight gain over time was consistent among all the treatment diets. The weight of the fish fed on diet BM33 was consistently higher after a month of feeding until the end of the experiment. Inclusion of BM above 67% (BM67) led to low weight gain especially after 1.5 months of feeding.

The weight gain of fish was significantly higher when fed on diet BM33. Further, the growth trend of fish fed the control diet was similar to those provided by diet BM100. The better performance of the fish consuming diet BM33 could be attributed to an adequate balance of essential nutrients. Our observation is supported by Khan [34] who demonstrated that fish fed balanced diets had excellent growth parameters probably due to good palatability, high digestibility and a balanced amino acid profile [34]. Furthermore, improved fish growth performance has also been reported when two or more animal protein sources are combined [35]. This is in accordance with our observation following the provision of diets containing 33% BM and 77% FM (diet BM33). 

Other studies involving the replacement of FM with BM have reported varying levels of favorable replacements. For example, Rana et al. [29] reported a better growth rate for a Nile tilapia fry fed diet with 50% inclusion of dehydrated BM as a substitute to FM followed by 25% BM inclusion. This is also further supported by Sealey et al. [21] who recommended a 50% BM inclusion in practical diets of rainbow trout. However, St-Hilaire et al. [20] showed that FM could be replaced by BM up to 25% in the rainbow trout (*Oncorhynchus mykiss*) diet, whereas Bondari and Sheppard [36] demonstrated that BM can replace up to 100% of the FM in the diets of catfish (*Ictalurus punctatus*) and blue tilapia (*Oreochromis aureus*).

A low FCR was observed for fish fed diet BM33, which directly translates to higher quality feed compared to others. Contrary to our results, Rana et al. [29] reported a lower FCR value (1.7) in fish diets following the replacement of FM with 50% BM. In the similar studies, the FCR was 1.91 following a 25% BM inclusion in the fish diet. However, diets with 100% substitution of FM by BM and 0% incorporation of BM (i.e., 100% FM) recorded FCR values of 2.26 and 2.25, respectively, which is consistent with the outcome of our study.

Survival rate (SR) is a crucial parameter when planning for an effective harvest. It provides useful information on feed rations and standing biomass in small and large aquaculture production systems [37]. However, the survival rate of the fish in the present study was not significantly different (*p* = 0.15) among the diet types (Table 4). The high survival rates observed in our experiments might be attributed to the high-quality feeds (Table 3) as well as proper management practices such as extended shelf-life or proper storage of feeds, good handling of fish during sampling and conducive physio-chemical parameters of pond water. All the water quality properties (Table 3) were within the standard values necessary for optimal production conditions for *Nile tilapia* [38].

The economics of partially or complete replacement of fishmeal with BM in Nile tilapia rations are presented in Table 5. The amount of feed, unit cost per kg of feed and overall fish biomass harvested reduced with increasing levels of BM as a substitute to FM (Table 5). Similarly, the profit index was observed to increase with increasing substitution of FM with BM. The estimated biomass value was highest in diet BM33 and lowest in BM100. However, diet BM100 recorded the highest profit index while the control diet had the least.

The unit price of formulation of the rations decreased gradually as fishmeal was substituted with BM, particularly in diet BM100, which had the lowest cost (USD 0.172 per fish) compared to the control diet (USD 0.201 per fish), which is equivalent to a 14.4% cost reduction. Previous studies have also reported a reduction in the cost of feed when BM was used to replace major conventional sources of proteins [39,40]. However, no statistically significant differences were observed; diet BM33 numerically had the highest CBR (2.172) and RoI (217.2) compared to other diets. These results indicate that replacing FM with BM would not only result in cheaper diets, but also better economic returns.

## 5. Conclusions

In the past decades, aquafeed millers have continued to manufacture tilapia fish feeds with FM inclusion levels of 20–250 g/kg of feed due to their high-quality nutrient availability [40]. In this study, it has been demonstrated that FM can be substituted with BM in *O. niloticus* diets up to 67% without compromising their growth quality parameters. Cognisant of the fact that the current market price of FM is high and very competitive due to its scarcity and regular bans following overfishing by many national governments in East Africa, substitutions with BM would significantly lower the cost of pelletized aquafeed production for tilapia with well-balanced nutrient composition. The RoI and CBR were similar among diet types, suggesting that BM is a cost-effective high-quality ingredient in compounded fish feed to grow tilapia fish to marketable size. Thus, our findings would inform policy makers to support BM integration into large scale commercial feed manufacturing and enhance sustainable intensification of aquaculture production, contributing significantly to food and nutritional security in the country.

## Figures and Tables

**Table 1:** Composition of ingredients in the experimental diets.

Ingredient	Control	BM33	BM67	BM100
Maize germ meal	25.0	25.0	25.0	25.0
Wheat pollard meal	42.8	35.4	28.1	17.9
FM	32.2	22.8	13.3	0.0
BM	0.0	16.8	33.6	57.1
Calculated quantity	100.0	100.0	100.0	100.0
Estimated nutrient composition				
Protein content (% DM)	30.3	30.0	29.7	29.5
Energy (KCal/kg DM)	3031.4	3099.6	3168.0	3263.6
Crude fat (% DM)	3.5	8.9	14.3	21.6
Crude fibre (% DM)	2.4	3.7	4.9	6.6

BM, black soldier fly larvae meal; FM, Fishmeal; Control—(100% FM; 0% BM); BM33—(67% FM; 33% BM); BM67—(33% FM; 67% BM) and BM100—(0% FM; 100% BM).

**Table 2:** Mean (± SE) pond water quality parameters during the rearing period of *Nile tilapia*.

Parameter	Mean Value	Optimal Range
pH	8.9 ± 0.20	6–9
Conductivity	108.0 ± 7.07	100–2000
Temperatures (°C)	26.8 ± 1.66	25–27
Total dissolved solids	53.8 ± 3.67	≥3
Salinity	0.1 ± 0.001	-
Phosphate (mg/L)	0.4 ± 0.01	-
Ammonia (mg/L)	0.1 ± 0.00	0.02–0.5
Nitrates (mg/L)	0.4 ± 0.00	0.2–2.19

**Table 3:** Mean (± SE) proximate composition of the experiment diets.

Parameter	Control	BM33	BM67	BM100	F-Value	*p*-Value
Dry matter	96.0 ± 0.33 ^a^	98.0 ± 0.28 ^a^	97.0 ± 0.32 ^a^	98.0 ± 0.45 ^a^	12.54	0.59
Ash	8.0 ± 0.24 ^a^	6.5 ± 0.31 ^a^	6.0 ± 0.11 ^a^	5.0 ± 0.26 ^a^	8.38	0.09
Crude protein	29.4 ± 0.12 ^a^	28.2 ± 0.16 ^a^	29.0 ± 0.22 ^a^	28.6 ± 0.34 ^a^	4.56	0.34
NDF	24.0 ± 0.33 ^a^	28.0 ± 0.21 ^a^	27.0 ± 0.16 ^a^	27.0 ± 0.24 ^a^	6.94	0.19
ADF	7.0 ± 0.06 ^a^	9.5 ± 0.32 ^a^	6.0 ± 0.17 ^a^	6.5 ± 0.11 ^a^	2.29	0.41
Ether extract	5.2 ± 0.01 ^a^	10.20 ± 0.19 ^b^	13.4 ± 0.09 ^b^	14.4 ± 0.43 ^b^	5.11	0.03

Means with the small same letter (a and b) superscript in the same row are not statistically significant at *p >* 0.05; NDF—Neutral detergent fibre; ADF—acid detergent fibre; Control—(100% FM; 0% BM); BM33—(67% FM; 33% BM); BM67—(33% FM; 67% BM) and BM100—(0% FM; 100% BM).

**Table 4:** Mean (± SE) growth parameters of growing *Nile tilapia* fish fed on the experimental diets.

Parameter	Control	BM33	BM67	BM100	F-Value	*p*-Value
Initial body weight (g)	35.3 ± 0.05 ^a^	35.1 ± 0.01 ^a^	35.4 ± 0.01 ^a^	35.2 ± 0.06 ^a^	8.32	0.89
Final body weight (g)	153.7 ± 7.17 ^a^	176.5 ± 12.74 ^b^	141.1 ± 17.56 ^a,c^	125.5 ± 9.29 ^c^	12.65	0.02
Daily weight gain (g)	0.9 ± 0.02 ^a^	1.0 ± 0.08 ^b^	0.8 ± 0.01 ^a,c^	0.7 ± 0.05 ^c^	14.54	0.01
Daily Feed Intake (g/day)	2.3 ± 0.03 ^a^	2.6 ± 0.02 ^b^	2.2 ± 0.08 ^c^	2.1 ± 0.04 ^c^	17.43	0.02
Feed Conversion Ratio	2.7 ^a^	2.1 ^a^	2.6 ^a^	2.9 ^a^	6.87	0.14
Survival rate	98.4 ± 0.04 ^a^	98.1 ± 0.06 ^a^	97.9 ± 0.02 ^a^	97.6 ± 0.05 ^a^	4.29	0.88

Means with the same small letter (a, b and c) superscript in the same row are not significantly different at *p* > 0.05; Control—(100% FM; 0% BM); BM33—(67% FM; 33% BM); BM67—(33% FM; 67% BM) and BM100—(0% FM; 100% BM).

**Table 5:** Economic analysis (means ± SE) of partial and complete substitution of fishmeal (FM) with black soldier fly larvae meal (BM) in Nile tilapia pelletized feeds.

Treatment	Control	BM33	BM67	BM100	F-Value	*p*-Value
Total feed cost USD/fish (C)	0.201 ± 0.002 ^a^	0.223 ± 0.004 ^b^	0.187 ± 0.001 ^a^	0.172 ± 0.005 ^c^	27.41	0.0001
Live weight at harvesting (g)	0.154 ± 0.007 ^a^	0.177 ± 0.006 ^b^	0.142 ± 0.008 ^c^	0.125 ± 0.007 ^d^	41.95	0.0001
Sale of fish (S) ^a^	0.615 ± 0.003 ^a^	0.706 ± 0.001 ^b^	0.568 ± 0.004 ^a^	0.502 ± 0.001 ^c^	42.08	0.0001
Gross profit margin (P) ^b^	0.414 ± 0.005 ^a^	0.483 ± 0.003 ^a^	0.381 ± 0.006 ^a,b^	0.329 ± 0.004 ^b^	14.95	0.0012
Cost Benefit Ratio (CBR) ^c^	2.076 ± 0.001 ^a^	2.172 ± 0.002 ^a^	2.043 ± 0.003 ^a^	1.917 ± 0.005 ^a^	0.79	0.5324
Return on Investment (RoI) ^d^	207.6 ± 0.003 ^a^	217.2 ± 0.006 ^a^	204.1 ± 0.004 ^a^	191.7 ± 0.003 ^a^	0.79	0.5323

Currency exchange was USD 1 at KSH). Mean values within rows, followed by the same small letter (a, b and c), are not significantly different at *p* < 0.05; Control—(100% FM; 0% BM); BM33—(67% FM; 33% BM); BM67—(33% FM; 67% BM) and BM100—(0% FM; 100% BM); ^a^ 4 USD/kg liveweight; ^c^ CBR = S/C; ^b^ P = S-C; ^d^ RoI = P/C × 100; over computation was based on the current market price of USD 4 per kg of tilapia fish.

## Data Availability

All relevant data are presented in the paper.
